# The Effect of Zinc on Post-neurosurgical Wound Healing: A Review

**DOI:** 10.7759/cureus.6770

**Published:** 2020-01-25

**Authors:** Dennis Adjepong, Saira Jahangir, Bilal Haider Malik

**Affiliations:** 1 Neurological Surgery, California Institute of Behavioral Neurosciences and Psychology, Fairfield, USA; 2 Neuroscience, California Institute of Behavioral Neuroscience and Psychology, Fairfield, USA; 3 Internal Medicine, California Institute of Behavioral Neurosciences and Psychology, Fairfield, USA

**Keywords:** zinc deficiency, delayed wound healing, zinc excess, trace mineral, mineral, micro-nutrient

## Abstract

The aim of this article is to explore neurosurgeons' knowledge and understanding of the physiology of zinc and provide current information about the role zinc plays in post-neurological wound healing. We review several medical journals and bring together the most updated information related to lesion-healing after surgery.

## Introduction and background

Monitoring and treating wounds in the post-neurosurgical procedure is crucial due to the high cost of treatment to the healthcare system in the US. Post-neurological wound-healing process could be complicated and complex due to the prevalence of deficiencies in anti-oxidants, trace minerals, vitamins, and micro-elements in patients [1].

Post-neurosurgical wound-healing involves a multilayered procedure administered by chronological steps plus inflammation, proliferation, and remodeling phases [2]. During the post-neurosurgical procedure, there is an exposure of the skull, dura mater, pia, arachnoid villi, sub-endothelium, and collagen. Collagen and material factors do trigger aggregation of the platelet, which results in chemokines release and endothelial developmental features to form fibrin lumping [3]. Neutrophil initially appears at the site of injury. It gets marginalized from the center of blood flow to the periphery. Neutrophil cleans debris and bacteria and provides good homeostasis for wound healing. Macrophage phagocytoses the bacteria and damaged tissue [4]. The inflammation phase does not usually exceed 96 hours.

The accretion of cells and tissues is considered to be the common factor that characterizes the proliferative stage. The post-neurosurgical wound at this phase includes keratinocytes, endothelial cells, and fibroblasts. A granulation tissue is formed, which is composed of extracellular matrix (ECM) and which replaces the fibrin clot. The ECM is formed from collagen, elastin, and proteoglycans [5]. There are many developmental factors and cytokines that participate in this phase, converting development factor-beta, vascular endothelial factor developmental, and interleukins (IL), which facilitates the angiogenesis process. This phase continues until the sixth week [6].

The makeover phase is the last stage in post-neurosurgical wound healing. It requires a good balance between the creation of new cells and apoptosis. Immature type III collagen, gradual degradation of ECM, and developed type I collagen are dangerous in this stage, which lasts for a considerably longer time [6,7]. Any deficiency in anti-oxidants, trace minerals, vitamins, and microelements impairs the lesion-healing process at this stage. While there have been a few comprehensive studies on monitoring and treatment of post-neurosurgical wound healing, this study aims to focus on the process of post-neurosurgical wound healing, monitoring, and how deficiencies in vitamins, antioxidants, trace minerals, and micronutrients play a vital role in compounding the problem. A huge part of this article will be dedicated to the role vitamins, anti-oxidants, trace nutrients, and micronutrients play in post-neurosurgical wound healing.

## Review

Our research methods involved reviewing and analyzing information available in medical journals about wound healing. The information gathered from healthcare journals was sorted out to develop a proper understanding of wound healing, including pathophysiology, biochemistry, and scientific analysis of wound healing. We hope to fill some of the gaps that exist in our understanding of wound healing and identify and address more gaps.

Our review and analysis of the journals have led us to conclude that deficiency of zinc is a prominent factor in delayed wound healing after neurological surgery [8]. We have sought to emphasize that many post-surgical wounds are not healing promptly due to zinc deficiency. Neurosurgeons around the world do not fully understand the wound-healing process and it has been neglected for far too long. Several published studies have clearly illustrated that zinc is a vital micronutrient for post-neurosurgical wound healing. 

The pathophysiology of zinc in wound healing

The essential micronutrient zinc plays a major role in wound healing. The human body contains less than 50 mg/kg of zinc. It is a key factor related to immune function, central nervous system, wound healing, and bone metabolism [8]. Zinc accounts for over 10% of DNA programmed by the human genome (~3,000 DNA/enzyme). Zinc-dependent DNA aids in gene transcription regulation, DNA repair, cell death, physiological processes, extracellular regulation, and antioxidant defense [9-13].

**Figure 1:**
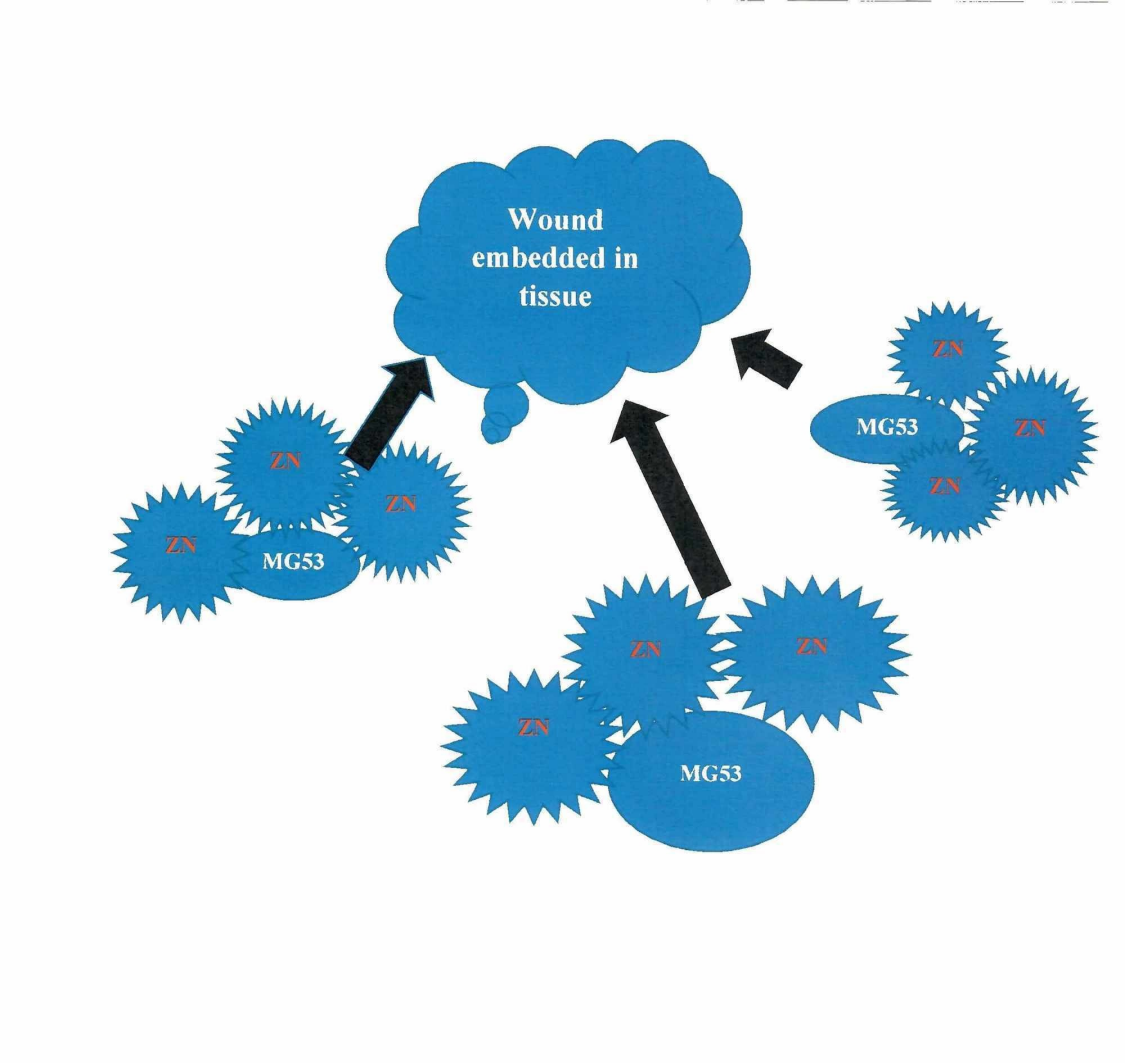
The mechanism of zinc in wound healing Zn: zinc; MG53: Mitsugumin 53

Zinc, a trace mineral, is found to be in low concentration in tissues and across cell membranes in post-neurosurgical wound-healing patients [8]. As such, zinc is firmly regulated through gene transcription rule, ion carriers, cellular homeostasis, and extracellular supplies [14]. During physiological processes, there is a small quantity of zinc in extracellular vesicles. The zinc transporter protein (ZIP) usually takes up zinc in intracellular vesicles [15,16]. Free zinc ions are found in the cytosol and have been identified as secondary messengers that are capable of targeting proteins to regulate numerous chemical and physiological pathways. Therefore, the availability of zinc and its regulation are essential components of cellular physiology [17,18]. The mechanism of action of zinc in wound healing is illustrated below (Figure 1).

Zinc deficiency is common in daily food intake. Dietary zinc is kept in human allium by a carrier-mediated process. The majority of zink found within the human body is in skeletal muscle (62%), followed by bone (29%), skin and liver (5%), and various other organs (2-3%) [19]. Due to the multilayered nature of zinc in our diets, the effects are extensive and involve several organ arrangements and matters. Globally, research dating back to 1970 has shown that zinc is a “common plight” to tissues [20]. Malnutrition is a known cause of zinc deficiency, and this has led to dietary problems that can manifest clinically as gastrointestinal (GI) malabsorption syndrome, liver and renal diseases, aging, immune dysfunction, mental and growth retardation, hypogonadism, and impaired healing of wounds [21-28]. Zinc shortage has been blamed for delays in wound healing [29,30]. Zinc deficiency plays a role in inflammation, mainly by elevating inflammatory response as well as causing damage to host tissue [31]. Zinc supplements have been given to post-neurosurgical and severely ill patients, patients with severe burn injury, hypodermic sore, insignificant surgery, and pressure ulcer [32-38]. Table 1 lays out statistical data, which have been discussed in other research studies, about the role zinc plays in wound healing.

**Table 1:** Studies that discuss zinc's role in wound healing

Number of studies	Author name	Year of publication	Country of origin of study	Findings
1	Cereda E, et al. [39]	2009	Italy	There was a significant reduction in the size of ulcers after 84 days of supplementation of zinc, arginine, anti-oxidants, and high protein formula ( 8-20mg zinc daily).
6	Wilkinson EA, et al. [9]	2012	UK	Zinc oxide paste-medicated dressing with a concentration between 6-15% for chronic venous leg ulcer improved wound healing.
1	Sakae K, et al. [40]	2013	Japan	A study of 42 patients with ulcers treated with zinc-containing polaprezinc versus oral L-carnosine (at 34 mg per day) showed no difference in healing.
1	Attia EA, et al. [10]	2014	Egypt	Ninety non-diabetics patients with uncomplicated wounds treated with 0.2 mg/100 mL per 10cm^2^ of zinc chloride solution reported significant improvement in wound healing.

The biochemistry of zinc in wound healing

Tripartite motif family (TRIM) proteins and an N-terminal ring zinc finger domain play important biochemical roles in regulating biochemical processes associated with wound healing and normal physiological processes. TRIM protein, Mitsugumin 53, and TRIM72 are implicated in tissue repair after injury. Vascular endothelial and transforming growth factors facilitate wound healing, and these growth factors require zinc for normal physiological functions [41,42]. The significant development of homeostasis is rapidly achieved when micronutrients are in the right proportions in the serum.

Economic implications

When wounds do not heal promptly, there is an increase in hospital visits; and it increases the burden on our healthcare insurance industry. Many patients become devastated and it affects their quality of life and economic prospects. Increased awareness about zinc supplements' ability to help heal wounds faster will expedite the treatment process and help reduce the occurrence of hospital visits after neurosurgical procedures.

Clinical implications

There is substantial evidence that without zinc a post-neurosurgical wound will take more time to heal. This touches on the value we place on ensuring patients a decent quality of life after surgery. Non-usage of zinc increases patient visits to the clinic and may lead to financial and mental distress. It will be beneficial for neurosurgeons to check the levels of antioxidants and zinc before surgery to help curb hospital re-admissions.

Scientific analysis

Of the many studies read, reviewed, and analyzed, the article by Pei-Hui Lin et al was a prominent source of reliable information because it presented detailed facts and scientific evidence to substantiate that zinc is critical to wound healing [8]. Neurosurgeons should be encouraged to use topical zinc ointment more often to help wounds heal faster.

Unanswered questions

Despite overwhelming evidence that delays in wound healing can be prominently caused by trace elements like zinc, other anti-oxidants like Vitamin A, C, and E have been implicated as well [43-46]. Many issues still remain unaddressed pertaining to the subject under review, such as the prevalence of inordinate delay in wound healing and the absence of reliable data on the economic burden it places on society in general and insurance industry in particular. There is insufficient scientific and statistical data involving the general population and sample size with regard to the role zinc plays in wound healing.

## Conclusions

Delayed wound healing after surgery has been the frontline worry for neurosurgeons in recent times. Whiles other anti-oxidants and trace elements like zinc are heavily implicated, zinc supplementation has proven to be an overwhelming success in managing this condition. Further studies are needed under rigorous conditions to substantiate the role zinc plays in wound healing after neurosurgical procedures.

## References

[1] Lindley LE, Stojadinovic O, Pastar I, Tomic-Canic M (2016). Biology and biomarkers for wound healing. Plast Reconstr Surg.

[2] Gauglitz GG, Korting HC, Pavicic T, Ruzicka T, Jeschke MG. (2011). Hypertrophic scarring and keloids: pathomechanisms and current and emerging treatment strategies. Mol Med.

[3] Berman B, Maderal A, Raphael B (2017). Keloids and hypertrophic scars: pathophysiology, classification, and treatment. Dermatol Surg.

[4] Su WH, Cheng MH, Lee WL, Tsou TS, Chang WH, Chen CS, Wang PH (2010). Nonsteroidal anti-inflammatory drugs for wounds: pain relief or excessive scar formation?. Media Inflamm.

[5] Plikus MV, Guerrero-Juarez CF, Ito M (2017). Regeneration of fat cells from myofibroblasts during wound healing. Science.

[6] Tsai HW, Wang PH, Tsui KH (2018). Mesenchymal stem cell in wound healing and regeneration. J Chin Med Assoc.

[7] Roohani N, Hurrell R, Kelishadi R, Schulin R (2013). Zinc and its importance for human health: an integrative review. J Res Med Sci.

[8] Lin PH, Sermersheim M, Li H, Lee PHU, Steinberg SM, Ma J (2020). Zinc in wound healing modulation. Nutrients.

[9] Wilkinson EA (2020). Oral zinc for arterial and venous leg ulcers. Cochrane Database Syst Rev.

[10] Attia EA, Belal DM, El Samahy MH, El Hamamsy MH (2019). A pilot trial using topical regular crystalline insulin vs. aqueous zinc solution for uncomplicated cutaneous wound healing; impact on quality of life. Wound Repair Regen.

[11] Cereda E, Gini A, Pedrolli C, Vanotti A (2019). Disease-specific, versus, standard, nutritional support for the treatment of pressure ulcer in institutionalized older adults: a randomized controlled trial. J Am Geriatr Soc.

[12] Zheng J, Lang Y, Zhang Q (2015). Structure of human MDM2 complexed with RPL11 reveals the molecular basis of p53 activation. Genes Dev.

[13] Cho JG, Park S, Lim CH (2020). ZNF224, Krüppel like zinc finger protein, induces cell growth and apoptosis-resistance by down-regulation of p21 and p53 via miR-663a. Oncotarget.

[14] Cronin L, Walton PH (2003). Synthesis and structure of [Zn(OMe)(L)] x Zn(OH)(L)] x 2(BPh4), L = cis,cis-1,3,5-tris[(E,E)-3-(2-furyl)acrylideneamino]cyclohexane: structural models of carbonic anhydrase and liver alcohol dehydrogenase. Chem Commun (Camb).

[15] Tomlinson ML, Garcia-Morales C, Abu-Elmagd M, Wheeler GN (2008). Three matrix metalloproteinases are required in vivo for macrophage migration during embryonic development. Mech Dev.

[16] Pawlak K, Mysliwiec M, Pawlak D (2012). The alteration in Cu/Zn superoxide dismutase and adhesion molecules concentrations in diabetic patients with chronic kidney disease: the effect of dialysis treatment. Diabetes Res Clin Pract.

[17] Choi S, Bird AJ (2014). Zinc’ing sensibly: controlling zinc homeostasis at the transcriptional level. Metallomics.

[18] Colvin RA, Holmes WR, Fontaine CP, Maret W (2010). Cytosolic zinc buffering and muffling: their role in intracellular zinc homeostasis. Metallomics.

[19] Hojyo S, Fukada T (2016). Zinc transporters and signaling in physiology and pathogenesis. Arch Biochem Biophys.

[20] Kambe T, Tsuji T, Hashimoto A, Itsumura N (2015). The physiological, biochemical, and molecular roles of zinc transporters in zinc homeostasis and metabolism. Physiol Rev.

[21] Bozeman RA, Chimienti F, Giblin LJ (2010). Free zinc ions outside a narrow concentration range are toxic to a variety of cells in vitro. Exp Biol Med (Maywood).

[22] Zhang T, Sui D, Hu J (2020). Structural insights of ZIP4 extracellular domain critical for optimal zinc transport. Nat Commun.

[23] Frederickson CJ, Manton WI, Frederickson MH, Howell GA, Mallory MA (1982). Stable-isotope dilution measurement of zinc and lead in rat hippocampus and spinal cord. Brain Res.

[24] Frederickson CJ, Moncrieff DW (1994). Zinc-containing neurons. Biol Signals.

[25] Giroux EL, Henkin RI (1972). Competition for zinc among serum albumin and amino acids. Biochim Biophys Acta.

[26] Gosbell A, Favilla I, Jablonski P (1996). The effects of insulin on the electroretinogram of the bovine retina in vitro. Curr Eye Res.

[27] Hambidge KM (1981). Zinc deficiency in man: its origins and effects. Philos Trans R Soc Lond B Biol Sci.

[28] Tran CD, Katsikeros R, Manton N, Krebs NF, Hambidge KM, Butler RN, Davidson GP (2011). Zinc homeostasis and gut function in children with celiac disease. Am. J. Clin. Nutr.

[29] Martinez SS, Campa A, Li Y, Fleetwood C, Stewart T, Ramamoorthy V, Baum MK (2017). Low plasma zinc is associated with higher mitochondrial oxidative stress and faster liver fibrosis development in the Miami Adult Studies in HIV cohort. J Nutr.

[30] Mariani E, Mangialasche F, Feliziani FT (2008). Effects of zinc supplementation on antioxidant enzyme activities in healthy old subjects. Exp Gerontol.

[31] Gammon NZ, Rink L (2020). Zinc in infection and inflammation. Nutrients.

[32] Mohammed J, Mehrotra S, Schulz H, Lim R (2017). Severe infant rash resistant to therapy due to zinc deficiency. Pediatr Emerg Care.

[33] Hagmeyer S, Haderspeck JC, Grabrucker AM (2020). Behavioral impairments in animal models for zinc deficiency. Front Behav Neurosci.

[34] Boycott KM, Beaulieu CL, Kernohan KD (2015). Autosomal-recessive intellectual disability with cerebellar atrophy syndrome caused by mutation of the manganese and zinc transporter gene SLC39A8. Am J Hum Genet.

[35] Rotter I, Kosik-Bogacka DI, Dolegowska B, Safranow K, Kuczyńska M, Laszczyńska M (2016). Analysis of the relationship between the blood concentration of several metals, macro- and micronutrients and endocrine disorders associated with male aging. Environ Geochem Health.

[36] Zorrilla P, Gómez LA, Salido JA, Silva A, López-Alonso A (2006). Low serum zinc level as a predictive factor of delayed wound healing in total hip replacement. Wound Repair Regen.

[37] Lansdown AB, Mirastschijski U, Stubbs N, Scanlon E, Agren MS (2007). Zinc in wound healing: theoretical, experimental, and clinical aspects. Wound Repair Regen.

[38] Wang K, Zhou B, Kuo YM, Zemansky J, Gitschier J (2002). A novel member of a zinc transporter family is defective in acrodermatitis enteropathica. Am J Hum Genet.

[39] Cerada E, Gini A, Pedrolli C, Vanotti A (2009). Disease-specific, versus, standard, nutritional support for the treatment of pressure ulcer in institutionalized older adults: a randomized controlled trial. J Am Geriatr Soc.

[40] Sakae K, Agata T, Kamide R, Yanagisawa H (2013). Effects of L-carnosine and its zinc complex (Polaprezinc) on pressure ulcer healing.. Nutr. Clin. Pract.

[41] Perafán-Riveros C, França LF, Alves AC, Sanches JA Jr (2002). Acrodermatitis enteropathica: case report and review of the literature. Pediatr Dermatol.

[42] Kogan S, Sood A, Garnick MS (2017). Zinc and wound healing: a review of zinc physiology and clinical applications. Wounds.

[43] Besecker BY, Exline MC, Hollyfield J, Phillips G, Disilvestro RA, Wewers MD, Knoell DL (2011). A comparison of zinc metabolism, inflammation, and disease severity in critically ill infected and noninfected adults early after intensive care unit admission. Am J Clin Nutr.

[44] Adjepong M, Agbenorku P, Brown P, Oduro I (2016). The role of antioxidant micronutrients in the rate of recovery of burn patients: a systematic review. Burns Trauma.

[45] Rech M, To L, Tobin A, Smoot T, Mlynarek M (2014). Heavy metal in the intensive care unit: a review of current literature on trace element supplementation in critically ill patients. Nutr Clin Pract.

[46] Kurmis R, Greenwood J, Aromataris E (2016). Trace element supplementation following severe burn injury: a systematic review and meta-analysis. J Burn Care Res.

